# Early Life Exposure to Chronic Intermittent Hypoxia Primes Increased Susceptibility to Hypoxia-Induced Weakness in Rat Sternohyoid Muscle during Adulthood

**DOI:** 10.3389/fphys.2016.00069

**Published:** 2016-03-04

**Authors:** Fiona B. McDonald, Eugene M. Dempsey, Ken D. O'Halloran

**Affiliations:** ^1^Health Sciences Centre, School of Medicine and Medical Science, University College DublinDublin, Ireland; ^2^Department of Paediatrics and Child Health, Cork University Maternity Hospital and the Irish Centre for Fetal and Neonatal Translational Research, University College CorkCork, Ireland; ^3^Department of Physiology, School of Medicine, University College CorkCork, Ireland

**Keywords:** apnea, developmental plasticity, respiratory muscle dysfunction, upper airway patency, neonatal

## Abstract

Intermittent hypoxia is a feature of apnea of prematurity (AOP), chronic lung disease, and sleep apnea. Despite the clinical relevance, the long-term effects of hypoxic exposure in early life on respiratory control are not well defined. We recently reported that exposure to chronic intermittent hypoxia (CIH) during postnatal development (pCIH) causes upper airway muscle weakness in both sexes, which persists for several weeks. We sought to examine if there are persistent sex-dependent effects of pCIH on respiratory muscle function into adulthood and/or increased susceptibility to re-exposure to CIH in adulthood in animals previously exposed to CIH during postnatal development. We hypothesized that pCIH would cause long-lasting muscle impairment and increased susceptibility to subsequent hypoxia. Within 24 h of delivery, pups and their respective dams were exposed to CIH: 90 s of hypoxia reaching 5% O_2_ at nadir; once every 5 min, 8 h per day for 3 weeks. Sham groups were exposed to normoxia in parallel. Three groups were studied: sham; pCIH; and pCIH combined with adult CIH (p+aCIH), where a subset of the pCIH-exposed pups were re-exposed to the same CIH paradigm beginning at 13 weeks. Following gas exposures, sternohyoid and diaphragm muscle isometric contractile and endurance properties were examined *ex vivo*. There was no apparent lasting effect of pCIH on respiratory muscle function in adults. However, in both males and females, re-exposure to CIH in adulthood in pCIH-exposed animals caused sternohyoid (but not diaphragm) weakness. Exposure to this paradigm of CIH in adulthood alone had no effect on muscle function. Persistent susceptibility in pCIH-exposed airway dilator muscle to subsequent hypoxic insult may have implications for the control of airway patency in adult humans exposed to intermittent hypoxic stress during early life.

## Introduction

The environment experienced during development can have long-lasting effects on structure and function of the respiratory system. Early life hypoxia is a common theme within the neonatal intensive care unit, primarily related to preterm infants. Altered oxygen availability during vulnerable periods of early life can ultimately induce persistent maladaptive outcomes in the respiratory control system (Bavis et al., 2004, 2010, 2011; Donnelly et al., 2005; Reeves et al., 2006; Prabhakar et al., 2007; Bavis and Mitchell, 2008; Mayer et al., 2013; Nanduri and Prabhakar, 2013). Perturbations in oxygen homeostasis during neonatal development can increase subsequent susceptibility to respiratory instability, thereby generating an underlying predisposition to the development of respiratory morbidity.

Intermittent hypoxia (IH) can be experienced during postnatal life in several different circumstances, notably apnea of prematurity (AOP), chronic lung diseases, and childhood obstructive sleep apnea (OSA) (Marshall and Wyche, 1972; Thach, 1985; Vuono et al., 2007; Katz, 2009; Pawar, 2012). McNamara and Sullivan (2000) have proposed that adult patients with sleep apnea may have been predisposed to developing apnea since early infancy. Indeed there is compelling evidence linking prematurity to increased risk of developing sleep-disordered breathing in childhood (Rosen et al., 2003; Hibbs et al., 2008; Raynes-Greenow et al., 2012), with one large-scale study revealing that diagnosis of sleep-disordered breathing is more prevalent in children born preterm, but not in those born small for gestational age (Raynes-Greenow et al., 2012). In broad terms, the long-term consequences of exposure to chronic IH during postnatal development (pCIH) on adult respiratory system function are understudied. Although the underlying mechanisms giving rise to apneas apparent in AOP, childhood OSA and adult OSA may differ, if untreated they all result in various patterns of exposure to chronic intermittent hypoxia (CIH), which has been shown in various animal models to be pivotal in the development, exacerbation, and perpetuation of various OSA-related co-morbidities, with resultant long-term adverse effects. Of relevance, exposure to CIH has been shown to alter the respiratory control system at multiple levels including aberrant remodeling in the peripheral sensors, medullary rhythm and pattern generators, and efferent motor pathways (Peng et al., 2003, 2004; Dick et al., 2007; Julien et al., 2008; Edge et al., 2012a).

Recently we reported that pCIH causes sternohyoid muscle impairment, which persists for at least 3 weeks upon return to a normoxic environment (McDonald et al., 2015b). Consistent with the notion of unique critical periods during development for respiratory-related maladaptive plasticity, we have demonstrated that the same CIH exposure does not significantly alter sternohyoid muscle force in adult rats (McDonald et al., 2014), notwithstanding that more frequent and/or intense exposures to CIH cause sternohyoid and diaphragm muscle dysfunction (McGuire et al., 2002; Pae et al., 2005; Shortt et al., 2014). The sternohyoid is one of several pharyngeal dilator muscles that play a pivotal role in the control of airway caliber. Phasic contraction of the sternohyoid—an infrahyoid muscle—during inspiration, in concert with suprahyoid muscles such as the geniohyoid, displaces the hyoid bone ventrally (anteriorly) and in this way the sternohyoid functions as a pharyngeal dilator muscle, complementing the actions of other major airway dilators such as the genioglossus (Roberts et al., 1984; Van De Graaff et al., 1984; Strohl et al., 1987; Van Lunteren et al., 1987a,b, 1989). The sternohyoid displays phasic inspiratory activity in the rat (O'Halloran et al., 2002) and is recruited during hypoxia and hypercapnia (O'Halloran et al., 2002), and during obstructive airway events (Edge et al., 2012b).

Pharyngeal dilator muscle dysfunction in humans increases the propensity for airway collapse, with relevance to AOP and OSA. Therefore, we posit that pCIH-induced sternohyoid muscle weakness could serve to increase the risk of airway collapse in mammals, such as humans, with collapsible airways (particularly during sleep when airway tone decreases considerably). Thus, it is plausible to consider that early life exposure to CIH might result in persistent respiratory muscle dysfunction and/or increased susceptibility to subsequent stressors presenting later in life. The long-term consequences of pCIH on respiratory system function during adulthood are generally understudied, and there is a paucity of information concerning the effects of early life exposure to CIH on respiratory muscle function into adulthood, when many new challenges to respiratory homeostasis can often present, including alterations in the mechanics of the upper airway and neuromodulatory mechanisms controlling airway patency. We speculated that IH stress during postnatal development would cause persistent impairment in respiratory muscle function into adulthood. Moreover, we reasoned that animals exposed to pCIH stress would show increased susceptibility to CIH exposure presented again later in adult life.

## Methods

### Ethical approval

All protocols described in this study were performed under license from the Irish Government, Department of Health and Children in accordance with National and European legislation following institutional ethics committee approval.

### CIH exposure

**Figure 1:**
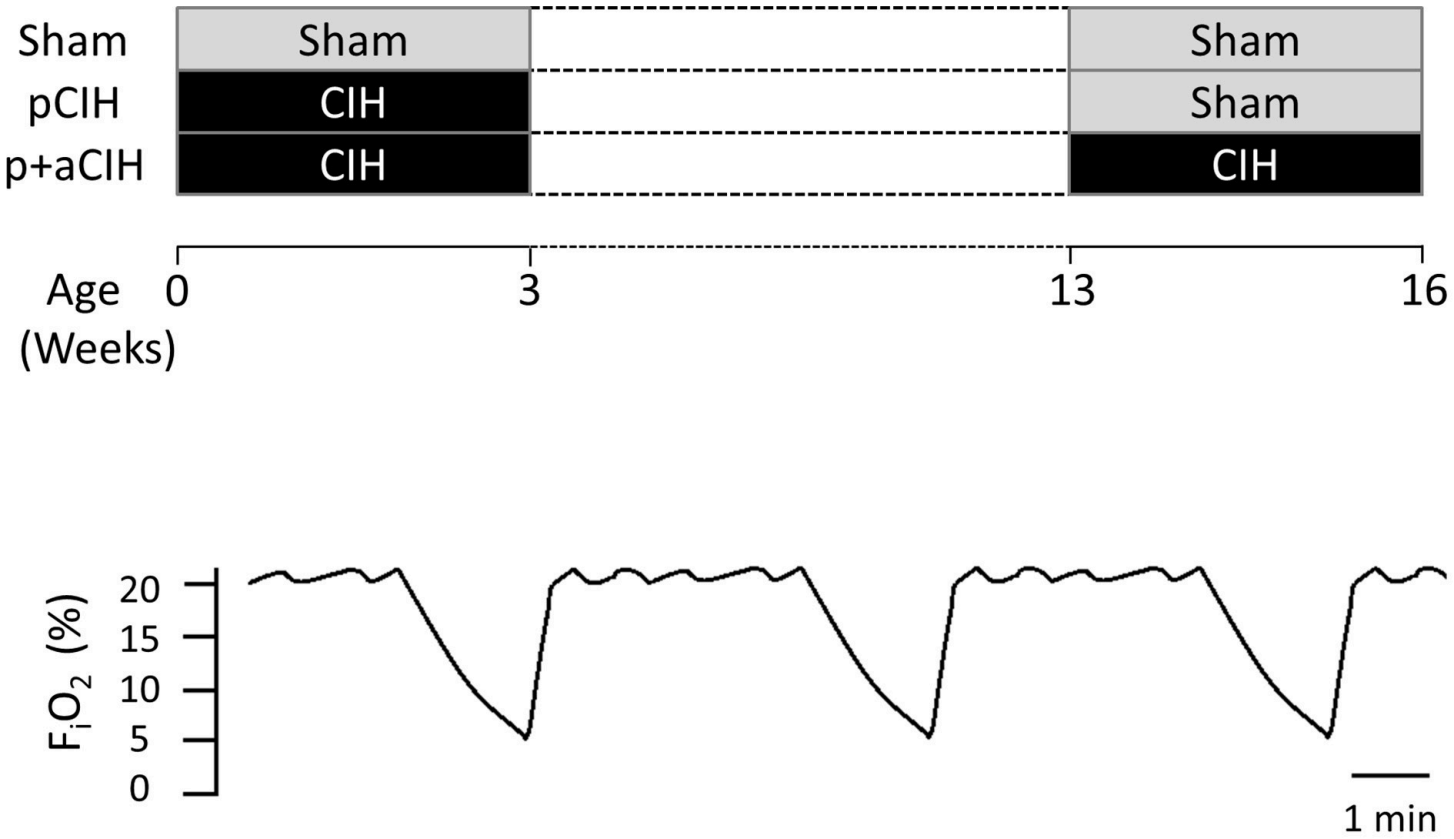
**Schematic showing the experimental groups highlighting the timing of gas exposures**. The lower panel shows a representative trace showing the CIH paradigm that animals were exposed to during early life and/or adulthood.

**Figure 2:**
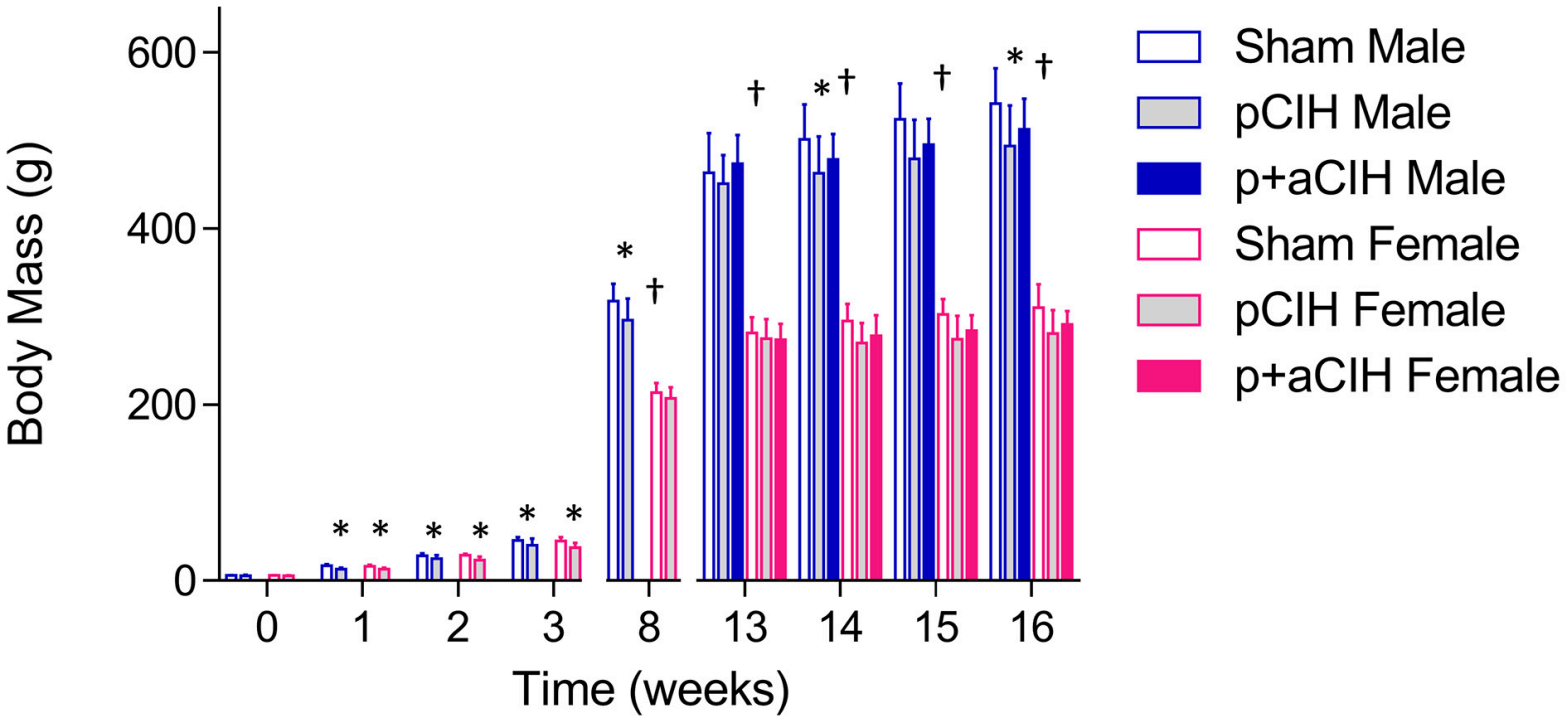
**Group data (mean ± SD) for body mass in male and female animals in all groups**. Data were analyzed by two-way (gas × sex) ANOVA. ^*^Indicates significant difference from corresponding sham value (*p* < 0.05). ^†^Indicates significant difference from male values (*p* < 0.05); SIdak *post-hoc* test.

A schematic of the study design is shown in Figure 1. Pregnant female Wistar rats were randomly divided into two groups: pCIH (*n* = 5) and sham (*n* = 3). Gas exposure of each dam and her respective litter began within 24 h of birth. The CIH-exposed animals were housed conventionally in standard cages placed in environmental chambers (*Oxycyler™*; Biospherix, NY, USA), wherein animals were exposed to 90 s of hypoxia (5% O_2_ at the nadir) followed by 210 s of normoxia (21% O_2_) i.e., 12 cycles per hour (see Figure 1). The hypoxia/re-oxygenation profile was run for eight consecutive hours during the light cycle. CIH exposure was performed daily for 3 weeks. The sham groups were exposed to a continuous normoxic environment in the same room. Animals had access to food and water *ad libitum.* Cages were routinely cleaned and food and water was replenished every second day. Animals were sexed from birth and body mass of the pups was monitored throughout the study (Figure 2).

Following gas exposures, male (*n* = 27) and female (*n* = 25) pups were separated and assigned to one of three groups: sham, pCIH, and pCIH+adult CIH (p+aCIH). Thereafter, all groups remained in normoxia in the same general environment with routine care for 10 weeks. At week 13, the p+aCIH group were re-exposed to CIH (same paradigm as described above) for a further 3 weeks. Sham and pCIH groups were exposed to normoxia in the same room during this period. All pCIH and p+aCIH rats were studied the day after the completion of the adult CIH exposure period.

In separate trials, we examined the effects of CIH exposure (same paradigm as described above) on adult female sternohyoid and diaphragm muscle [sham (*n* = 8) vs. CIH (*n* = 8)] to complement and extend our previous study in adult male rats demonstrating that the CIH paradigm described above has no effect on adult sternohyoid muscle function (McDonald et al., 2014).

### Experimental protocol

Rats were weighed, anesthetized with 5% isoflurane and animals were killed by cervical dislocation. Sternohyoid muscles were excised and cut into at least two longitudinal strips. Diaphragm muscle was also excised and cut into strips including rib and central tendon. Tissue bundles were suspended vertically with fine (non-elastic) string; one end of each strip was mounted to tissue holders, while the other end was tied firmly to a hook, which was connected to an isometric force transducer. The muscle fiber bundles affixed to the tissue holders were then suspended in standard water-jacketed tissue baths. The tissue baths open to atmosphere were filled with Krebs salt solution, maintained at 35°C and bubbled with 95% O_2_ and 5% CO_2_ under control conditions (or with 95% N_2_ and 5% CO_2_ in separate studies to generate acute severe hypoxic stress). The Krebs solution contained: 120 mM NaCl, 25 mM NaHCO_3_, 12 mM MgSO_4_,1.2 mM NaH_2_PO4, 2.5 mM CaGluconate, 5 mM KCl, and 11.5 mM Glucose. D-tubocurarine (25 μM) was used in all experiments to exclude any involvement of excitation of intramuscular nerve branches. The muscles were stimulated using supramaximal square wave constant current stimulators (S48 Stimulator, GRASS, Warwick, RI, USA), delivered via two platinum electrodes which flanked the tissue in the bath. The change in tension was transduced, amplified and converted from an analog-to-digital signal where it was displayed and recorded using Chart software (AD Instruments) on a computer for later analysis. Optimum muscle length (L_o_) was determined during repeated twitch stimulation while adjusting the length of the muscle with a micropositioner. The muscles were allowed to equilibrate for 10 min before starting the experimental protocol.

### Protocol

Single twitch was evaluated by measuring the single twitch tension (Pt), contraction time (CT), and half-relaxation time (HRT). Force-frequency relationship was determined by sequentially stimulating the muscle at 10, 20, 30, 40, 60, 80, and 100 Hz (300 ms train duration) allowing a 1 min interval between stimulation. Peak tetanic force was determined at 100 Hz stimulation. Ten minutes following the force-frequency protocol, fatigue was assessed in response to repeated tetanic contractions (40 Hz, 300 ms train duration) every 2 s for 5 min. This is a well-established protocol for the study of respiratory muscle (Cantillon and Bradford, 1998, 2000; O'Halloran, 2006; McMorrow et al., 2011; Skelly et al., 2011, 2012; McDonald et al., 2015a,b). At the end of the experiment, the L_o_ and weight of each muscle blotted dry was recorded in order to calculate cross-sectional area. The cross-sectional area of each strip was estimated by dividing the muscle mass (weight in grams) by the product of muscle L_o_ (in cm) and muscle density (assumed to be 1.06 g/cm^3^).

### Data analysis

For isolated muscle studies, single twitch and peak tetanic force (determined at 100 Hz stimulation) was calculated in N/cm^2^ of muscle cross-sectional area (CSA). Force-frequency relationship was determined by expressing force at each stimulus frequency as a % of peak force developed during the trial. Slope and EF_50_ values (i.e., stimulus frequency producing 50% of peak force) for sternohyoid and diaphragm muscle in sham and CIH-exposed muscles were determined. Fatigue was determined during the repeated muscle stimulation trials as amplitude of final contraction / amplitude of initial contraction × 100 i.e., % of control (initial) force. Data were compared statistically by two factor (gas × sex) analysis of variance (ANOVA). Two factor (gas × frequency) ANOVA was used for statistical judgment of force-frequency relationships; where appropriate, Sidak *post-hoc* tests were performed with *P* < 0.05 chosen as the criterion for significance in all tests. All data are presented as mean ± SD. Statistical tests were performed using GraphPad Prism 6 (Graph Pad Software, CA, USA).

## Results

### Body mass, hematocrit, and cardiac mass

Data are shown in Table 1 and Figure 2. Exposure to pCIH decreased body mass at age 16 weeks in male rats compared with corresponding sham controls. There was no effect of exposure to CIH on hematocrit, right, and left cardiac ventricle mass (Table 1).

**Table 1:** **Group data for body mass (BM); hematocrit (HCT); right ventricular mass (RV); left ventricular mass (LV); and right ventricular mass: left ventricular mass ratio (RV/LV) (all expressed as mean ± SD) in sham and CIH-exposed male and female rats**.

	**Male**			**Female**			**Two-way ANOVA (sex × gas)**
	**Sham (*n* = 8)**	**pCIH (*n* = 11)**	**p+aCIH (*n* = 8)**	**Sham (*n* = 8)**	**pCIH (*n* = 9)**	**p+aCIH (*n* = 8)**	
BM (g)	523 ± 28	473 ± 52^*^	505 ± 37	315 ± 20^†^	281 ± 20^†^	285 ± 19^†^	Gas: *P* = 0.0025; Sex: *P* < 0.0001; I: *P* = 0.43
Hct (%)	47 ± 6	44 ± 5	49 ± 4	40 ± 3^†^	43 ± 3	44 ± 3	Gas: *P* = 0.10; Sex: *P* < 0.0011; I: *P* = 0.0618
RV (mg)	260 ± 30	250 ± 30	270 ± 40	180 ± 20^†^	190 ±20^†^	190 ± 20^†^	Gas: *P* = 0.40; Sex: *P* < 0.0001; I: *P* = 0.5558
LV (mg)	920 ± 70	910 ± 60	940 ± 70	630 ± 40^†^	610 ± 20^†^	630 ± 70^†^	Gas: *P* = 0.51; Sex: *P* < 0.0001; I: *P* = 0.915
RV/LV	0.28 ± 0.03	0.27 ± 0.03	0.29 ± 0.03	0.29 ± 0.05	0.30 ± 0.03	0.30 ± 0.04	Gas: *P* = 0.78; Sex: *P* = 0.10; I: *P* = 0.56

Data were analyzed by two-way (gas × sex) ANOVA.

* Indicates significant difference from corresponding sham value; Sidak post-hoc test (p < 0.05).

† Indicates significant difference from corresponding male value; Sidak post-hoc test (p < 0.05).

### Single twitch force and contractile kinetics

**Figure 3:**
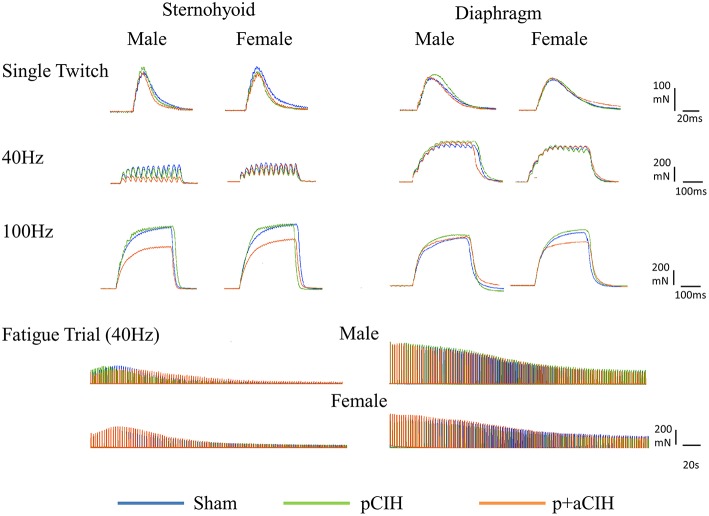
**Original representative traces from male and female sternohyoid (left panels) and diaphragm (right panels) muscle preparations from each experimental group: sham (blue), pCIH (green), and p+aCIH (red)**. Traces are superimposed to illustrate representative group effects. Peak tetanic force is achieved during 100Hz stimulation. Note the relative depression of force in p+aCIH sternohyoid (but not diaphragm) compared with sham control.

Sternohyoid and diaphragm muscle single twitch force (Pt) and contractile kinetics for sham, pCIH-, and p+aCIH-exposed male and female rats are shown in Figure 3 and Table 2. Pt was not significantly different between the three gas exposure groups in both male and female sternohyoid and diaphragm muscle. Similarly, contraction time and half-relaxation time were not significantly different between groups for both muscles in both sexes (Table 2).

**Table 2:** **Group data (mean ± SD) for peak twitch force (Pt), contraction time (CT), half-relaxation time (HRT), optimal length (Lo), and bundle cross-sectional area (CSA) of sham and CIH-exposed male and female sternohyoid and diaphragm muscle preparations examined under control conditions *ex vivo***.

	**Male**			**Female**			**Two-way ANOVA (sex × gas)**
	**Sham (*n* = 7)**	**pCIH (*n* = 8)**	**p+aCIH (*n* = 8)**	**Sham (*n* = 8)**	**pCIH (*n* = 7)**	**p+aCIH (*n* = 8)**	
**STERNOHYOID**							
Pt (N/cm^2^)	5.3 ± 1.1	4.8 ± 1.0	4.4 ± 0.6	4.6 ± 0.7	4.0 ± 0.9	4.5 ± 0.7	Gas: *P* = 0.12; Sex: *P* < 0.08; I: *P* = 0.25
CT (ms)	14 ± 1	14 ± 1	13 ± 1	14 ± 1	14 ± 2	14 ± 1	Gas: *P* = 0.26; Sex: *P* = 0.23; I: *P* = 0.062
HRT (ms)	12 ± 2	14 ± 4	10 ± 3	12 ± 2	13 ± 1	13 ± 3	Gas: *P* = 0.39; Sex: *P* = 0.29; I: *P* = 0.25
Lo (cm)	2.1 ± 0.2	2.0 ±0.1	2.0 ± 0.2	2.0 ±0.2	1.9 ±0.1	1.9 ± 0.2	Gas: *P* = 0.47; Sex: *P* = 0.08; I: *P* = 0.99
CSA (cm^2^)	0.04 ± 0.006	0.04 ± 0.01	0.05 ± 0.008	0.04 ± 0.006	0.03 ± 0.004^†^	0.03 ± 0.005^†^	Gas: *P* = 0.32; Sex: *P* < 0.0001; I: *P* = 0.04
	**Sham (***n* = 8**)**	**pCIH (***n* = 11**)**	**p+aCIH (***n* = 7**)**	**Sham (***n* = 7**)**	**pCIH (***n* = 9**)**	**p+aCIH (***n* = 7**)**	
**DIAPHRAGM**							
Pt (N/cm^2^)	4.3 ± 1.1	5.0 ± 0.8	4.9 ± 0.9	4.7 ± 0.6	5.6 ± 1.4	4.9 ± 1.3	Gas: *P* = 0.07; Sex: *P* = 0.32; I: *P* = 0.78
CT (ms)	21 ± 2	22 ± 2	21 ± 4	23 ± 3	22 ± 5	24 ± 3	Gas: *P* = 0.95; Sex: *P* = 0.09; I: *P* = 0.36
HRT (ms)	30 ± 7	31 ± 6	31 ± 7	32 ± 7	33 ± 3	31 ± 8	Gas: *P* = 0.80; Sex: *P* = 0.31; I: *P* = 0.91
Lo (cm)	2.0 ± 0.3	1.9 ± 0.2	1.9 ± 0.2	1.8 ± 0.1	1.8 ± 0.2	1.7 ± 0.2	Gas: *P* = 0.70; Sex: *P* = 0.006; I: *P* = 0.34
CSA (cm^2^)	0.04 ± 0.006	0.04 ± 0.01	0.05 ± 0.008	0.04 ± 0.006	0.03 ± 0.004^†^	0.03 ± 0.005	Gas: *P* = 0.4; Sex: *P* = 0.006; I: *P* = 0.3

Data were analyzed by two-way (gas × sex) ANOVA.

† Indicates significant difference from corresponding male value; Sidak post-hoc test (p < 0.05).

### Peak tetanic force

**Figure 4:**
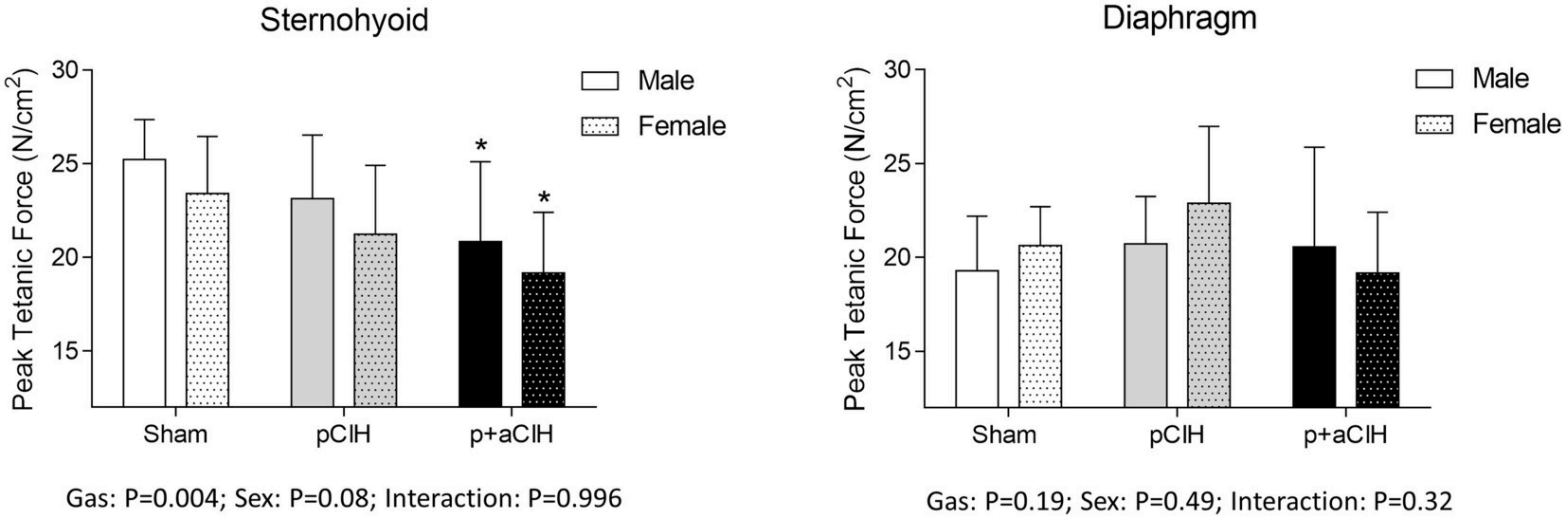
**Group data (mean ± SD) for sternohyoid and diaphragm muscle peak tetanic force in sham and CIH-exposed rats examined under control conditions *ex vivo***. Data were analyzed by two-way (gas × sex) ANOVA. ^*^Indicates significant difference from corresponding sham (*p* < 0.05); Sidak *post-hoc* test.

Representative traces are shown in Figure 3. Two way ANOVA (gas × sex) of sternohyoid muscle data sets revealed a significant gas effect (*P* = 0.004), with no significant independent sex effect and no interaction. *Post-hoc* analysis revealed a significant effect of p+aCIH exposure compared with sham both in male and female sternohyoid muscle (Figure 4). pCIH exposure did not significantly affect sternohyoid muscle force at age 16 weeks in both sexes. This reveals the principal finding of the study: pCIH exposure does not affect adult rat sternohyoid muscle force *per se*; however, a subsequent exposure to CIH in pCIH-exposed animals, results in a significant reduction in peak force-generating capacity compared with sham control, and this effect is of equal measure in both sexes.

Diaphragm peak tetanic force data are presented in Figure 4. In contrast to sternohyoid, diaphragm peak force was not significantly different across the groups.

### Force-frequency relationship

**Figure 5:**
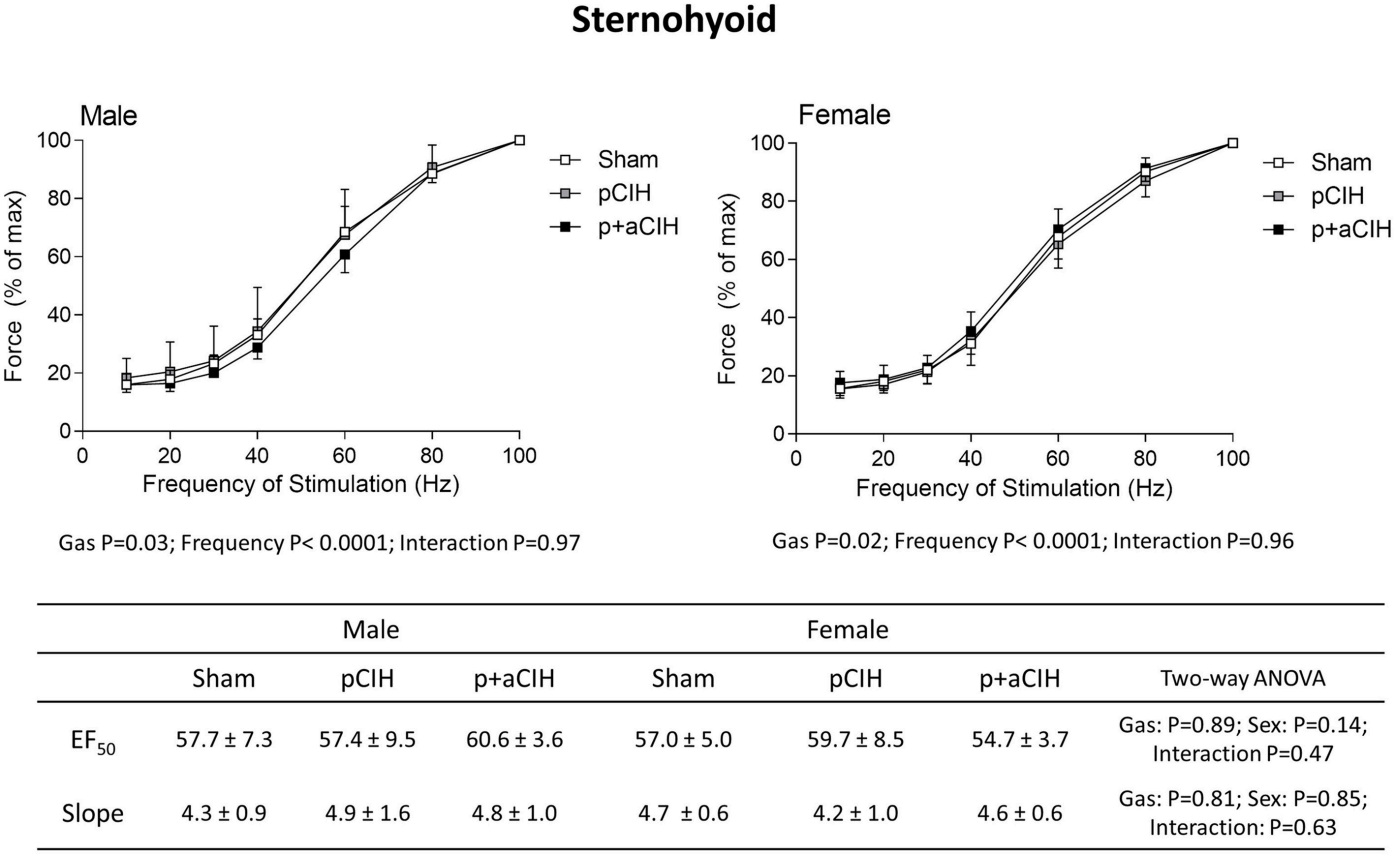
**Group data (mean ± SD) for force-frequency relationship of sternohyoid muscle in male and female sham and CIH-exposed rats examined under control conditions *ex vivo***. Data were analyzed by two-way (gas × frequency) ANOVA in male and female data sets. Table: Group data (mean ± SD) for EF_50_ and Hillslope of sternohyoid in male and female sham and CIH-exposed rats examined under control conditions *ex vivo*. Data were analyzed by two-way (gas × sex) ANOVA.

**Figure 6:**
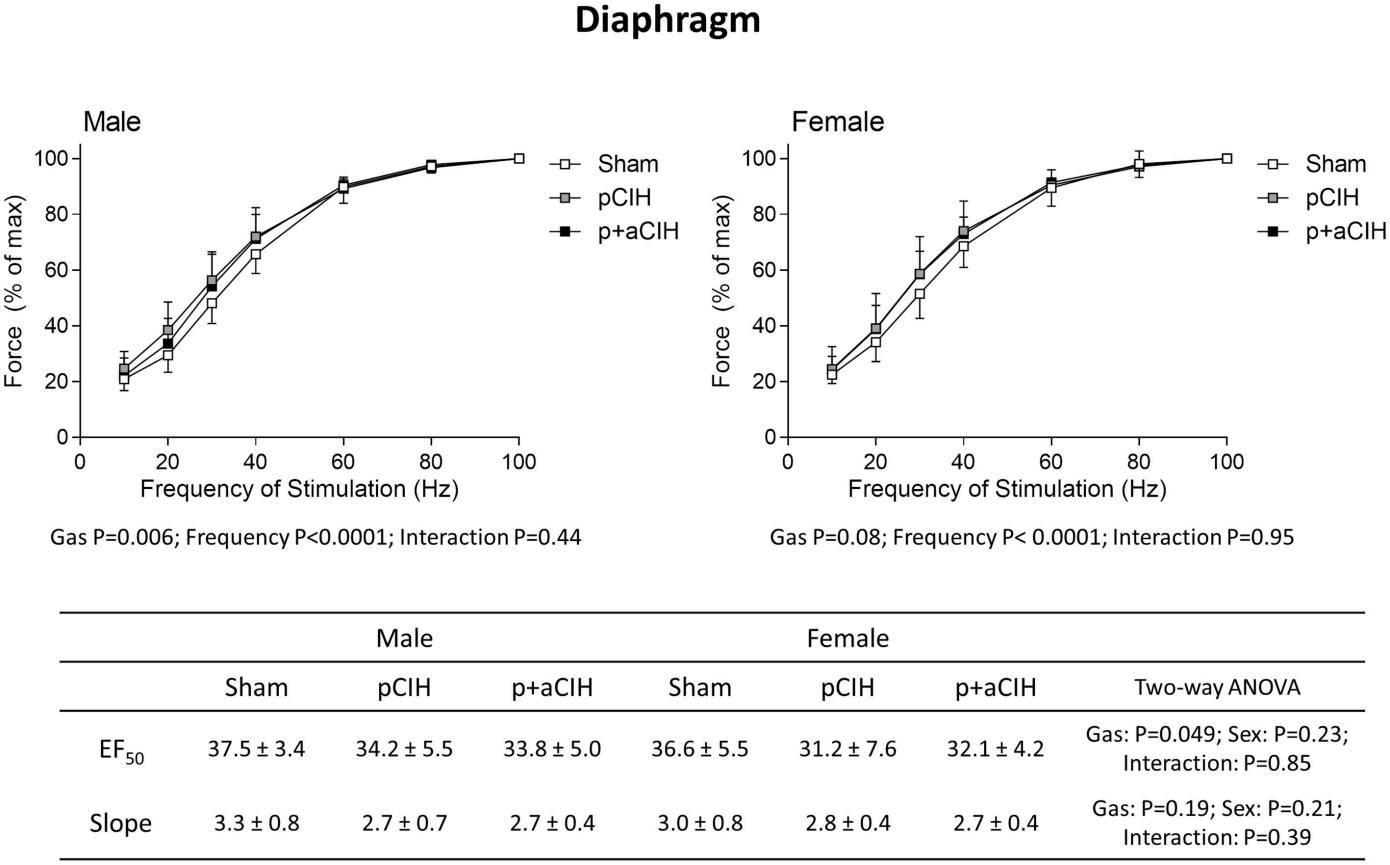
**Group data (mean ± SD) for force-frequency relationship of diaphragm muscle in male and female sham, pCIH-, and p+aCIH-exposed rats examined under control conditions *ex vivo***. Data were analyzed by two-way (gas × frequency) ANOVA in male and female data sets. Table: Group data (mean ± SD) for EF_50_ and Hillslope of diaphragm in male and female sham, and CIH-exposed rats examined under control conditions *ex vivo*. Data were analyzed by two-way (gas × sex) ANOVA.

Force-frequency relationships for sternohyoid (Figure 5) and diaphragm (Figure 6) muscles in all groups are shown. There was a significant rightward shift in the male sternohyoid force-frequency relationship (Figure 5). Conversely, there was a significant leftward shift in the male diaphragm force-frequency relationship (Figure 6). Data for Hillslope and EF_50_ between the three groups in both males and females are also presented.

### Fatigue

**Figure 7:**
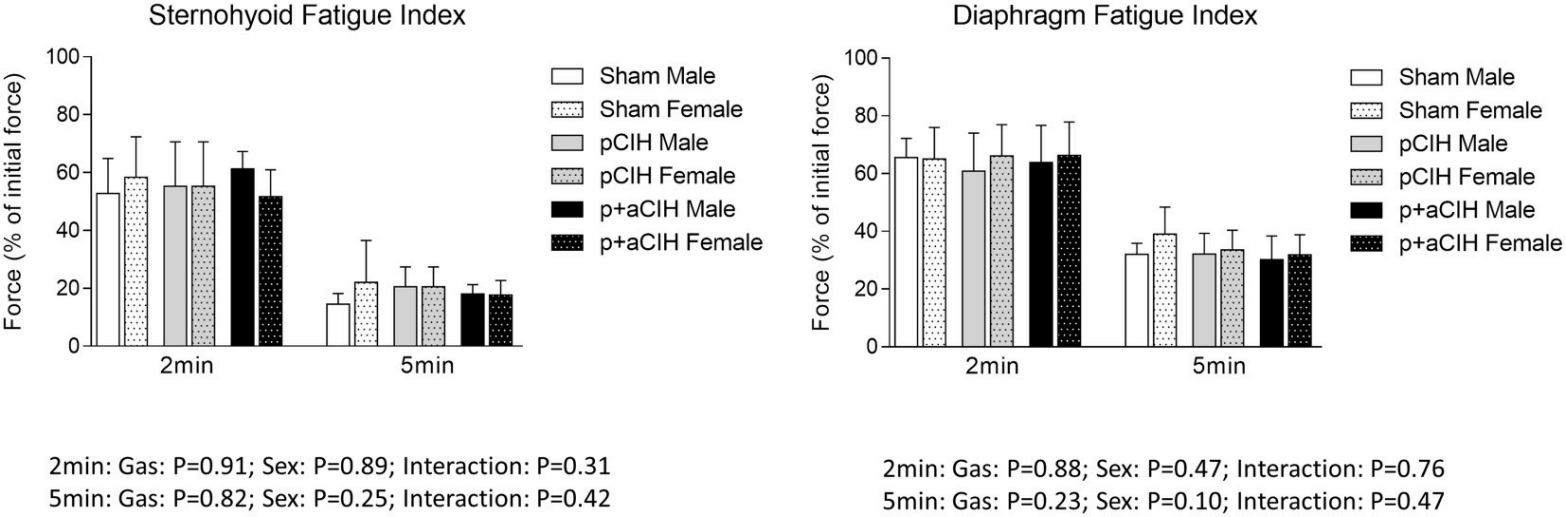
**Group data (mean ± SD) for fatigue index following 2 and 5 min of repeated muscle stimulation at 40 Hz in male and female sternohyoid and diaphragm muscle in sham and CIH-exposed rats examined under control conditions *ex vivo***. Data for force are expressed as a % of the initial (first) 40 Hz contraction during the fatigue trial. Data were analyzed by two-way (gas × sex) ANOVA.

Representative traces are shown in Figure 3. CIH exposure did not affect sternohyoid or diaphragm muscle endurance in male or female rats (Figure 7).

### Hypoxic tolerance

**Figure 8:**
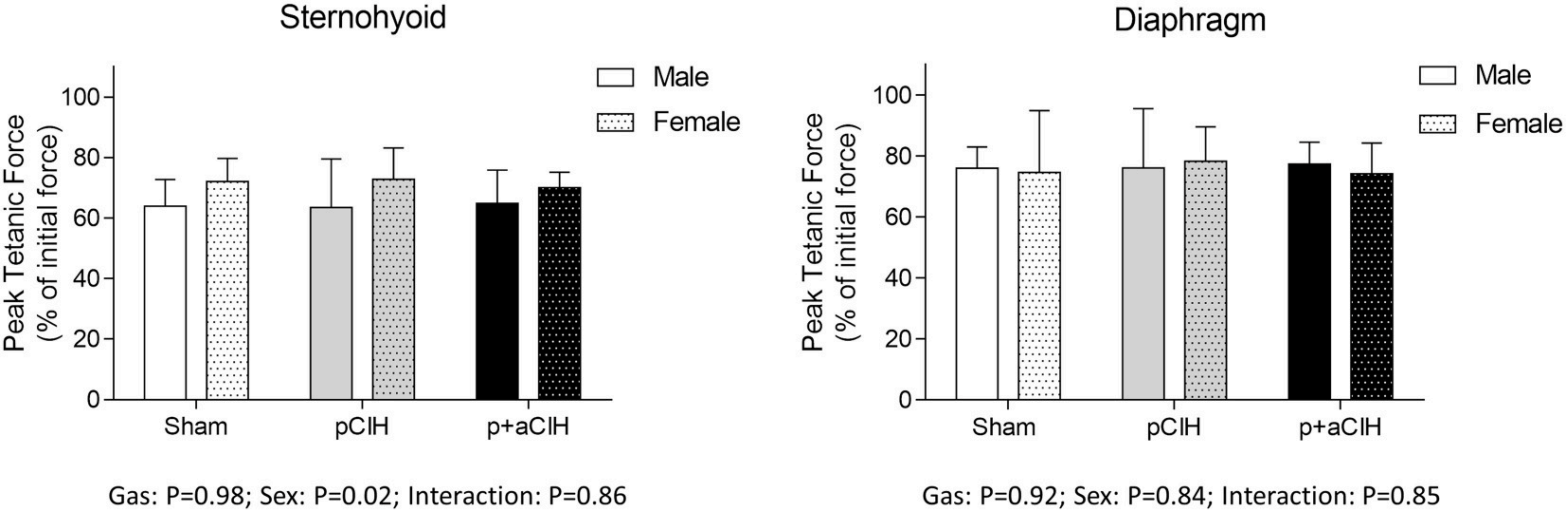
**Group data (mean ± SD) for sternohyoid and diaphragm muscle peak tetanic force in sham and CIH-exposed rats examined during acute severe hypoxic stress *ex vivo***. Data for force during hypoxia are expressed as a % of baseline (control) force determined in hyperoxia to illustrate the depressant effect of severe hypoxia on peak tetanic force at 100 Hz. Data were analyzed by two-way (gas × sex) ANOVA.

**Figure 9:**
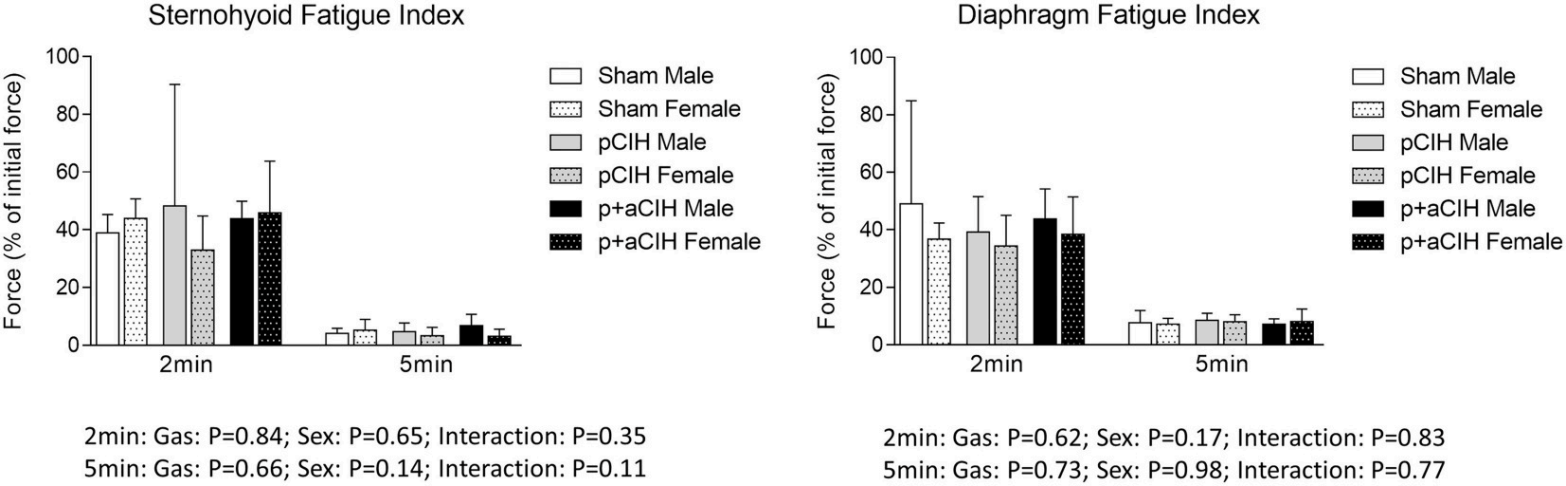
**Group data (mean ± SD) for fatigue index following 2 and 5 min of repeated muscle stimulation at 40 Hz in male and female sternohyoid and diaphragm muscle in sham and CIH-exposed rats examined during acute severe hypoxic stress *ex vivo***. Data for force are expressed as a % of the initial (first) 40 Hz contraction during the fatigue trial. Data were analyzed by two-way (gas × sex) ANOVA.

Sternohyoid and diaphragm muscle function was also assessed *ex vivo* in tissue baths gassed with 95% N_2_/5% CO_2_ to generate severe tissue hypoxia, which produced a loss of muscle strength compared with control preparations. However, there were no significant differences in contractile (Figure 8 and Table 3) and endurance (Figure 9) parameters comparing sham, pCIH, and p+aCIH muscles under conditions of severe hypoxia.

**Table 3:** **Group data (mean ± SD) for peak twitch force (Pt), contraction time (CT), half-relaxation time (HRT), optimal length (Lo), and bundle cross-sectional area (CSA) of sham and CIH-exposed male and female sternohyoid and diaphragm muscle preparations examined under acute severe hypoxic conditions *ex vivo***.

	**Male**			**Female**			**Two-way ANOVA (sex × gas)**
	**Sham (*n* = 8)**	**pCIH (*n* = 10)**	**p+aCIH (*n* = 8)**	**Sham (*n* = 8)**	**pCIH (*n* = 7)**	**p+aCIH (*n* = 8)**	
**STERNOHYOID**							
Pt (N/cm^2^)	3.0 ± 0.7	2.9 ± 0.7	2.6 ± 0.4	2.9 ± 0.6	3.2 ± 0.7	3.5 ± 0.7	Gas: *P* = 0.9 Sex: *P* = 0.07; I: *P* = 0.1
CT (ms)	12 ± 1	13 ± 3	13 ± 1	13 ± 1	12 ± 1	12 ± 1	Gas: *P* =;0.83 Sex: *P* = 0.32, I: *P* = 0.32
HRT (ms)	8 ± 1	9 ± 2	7 ± 2	8 ± 2	8 ± 1	7 ± 1	Gas: *P* = 0.29; Sex: *P* = 0.55; I: *P* = 0.38
Lo (cm)	2.0 ± 0.4	2.1 ± 0.2	2.1 ± 0.1	2.0 ± 0.2	1.8 ± 0.1	1.9 ± 0.1	Gas: *P* = 0.22; Sex: *P* = 0.02; I: *P* = 0.23
CSA (cm^2^)	0.05 ± 0.008	0.04 ± 0.005^*^	0.05 ± 0.008	0.03 ± 0.006^†^	0.03 ± 0.006	0.03 ± 0.006^†^	Gas: *P* = 0.04; Sex: *P* < 00.1; I: *P* = 0.17
	**Sham (***n* = 8**)**	**pCIH (***n* = 9**)**	**p**+**aCIH (***n* = 7**)**	**Sham (***n* = 6**)**	**pCIH (***n* = 8**)**	**p**+**aCIH (***n* = 7**)**	
**DIAPHRAGM**							
Pt (N/cm^2^)	3.7 ± 1.1	3.8 ± 1.1	3.6 ± 1.1	3.6 ± 1.1	4.5 ± 0.9	4.4 ± 0.6	Gas: *P* = 0.39; Sex: *P* = 0.17; I: *P* = 0.47
CT (ms)	17 ± 1	18 ± 2	17 ± 3	17 ± 2	19 ± 4	17 ± 2	Gas: *P* = 0.12; Sex: *P* = 0.28; I: *P* = 0.75
HRT (ms)	14 ± 2	16 ± 3	15 ± 4	15 ± 4	17 ± 3	17 ± 2	Gas: *P* = 0.17; Sex: *P* = 0.19; I: *P* = 0.93
Lo (cm)	1.9 ± 0.2	1.8 ± 0.2	1.9 ± 0.1	1.8 ± 0.3	1.8 ± 0.1	1.9 ± 0.3	Gas: *P* = 0.62; Sex: *P* = 0.51 I: *P* = 0.68
CSA (cm^2^)	0.04 ± 0.018	0.03 ± 0.016	0.04 ± 0.012	0.03 ± 0.009	0.03 ± 0.009	0.03 ± 0.005	Gas: *P* = 0.12; Sex: *P* = 0.007; I: *P* = 0.87

Data were analyzed by two-way (gas × sex) ANOVA.

* Indicates significant difference from corresponding sham value; Sidak post-hoc test (p < 0.05).

† Indicates significant difference from corresponding male value; Sidak post-hoc test (p < 0.05).

### Effects of CIH exposure during adulthood on sternohyoid and diaphragm muscle function

Exposure to CIH during adult life had no significant effect on contractile and endurance properties of the sternohyoid and diaphragm muscle of female rats (data not shown), consistent with our previous observation in male rats (McDonald et al., 2015b).

## Discussion

Despite the clinical relevance, the long-term effects of antecedent early life hypoxia on respiratory control are not well understood. This study was undertaken to investigate if there are persistent effects of pCIH exposure on respiratory muscle into adulthood. The data reveal that pCIH-induced sternohyoid muscle impairment described previously by our group (McDonald et al., 2015b) is apparently fully “recovered” by adulthood, insofar as when examined at 16 weeks, sternohyoid (and diaphragm) muscle is apparently not functionally different from age-matched sham exposed animals. However, of interest, pCIH-exposed sternohyoid muscle (but not diaphragm) retains an inherent susceptibility to subsequent hypoxic insult, such that when pCIH pre-conditioned muscle is re-exposed to CIH in adulthood (which itself has no effect on adult muscle (McDonald et al., 2014; this study), sternohyoid muscle weakness is revealed.

Studies that have examined the long-term ventilatory effects of postnatal intermittent hypoxia in rodents have shown persistent increases in normoxic ventilation and basal phrenic burst frequency, but blunted phrenic long-term facilitation (a form of respiratory neuroplasticity)and hypoxic ventilatory responses (Moss and Laferrière, 2006; Reeves et al., 2006). There is also evidence of long-lasting alterations induced by postnatal hypoxia on baroreflex function (Soukhova-O'hare et al., 2006). The present study is the first to examine the long-term effects of pCIH exposure on respiratory muscle function. Early life adversity can reportedly affect muscle function and metabolism. Some studies suggest that individuals born small for gestational age have reduced muscle mass (Phillips, 1995; Hediger et al., 1998; Gale et al., 2001; Sayer et al., 2004; Yliharsila et al., 2007) and impaired muscle strength in later life (Sayer et al., 1998, 2004; Kuh et al., 2002, 2006), which consequently increases disability and frailty. Furthermore, Jensen et al. (2007) showed, in a small cohort, that low birth weight altered subsequent skeletal muscle fiber composition and size.

We examined respiratory muscle responses to acute severe hypoxic challenge following CIH exposure in our model and observed no effect of CIH preconditioning on the depressant effects of acute severe hypoxia on sternohyoid and diaphragm muscle contractile and endurance properties. In recent years there is growing support for the “origin of adult disease in early life” hypothesis (De Boo and Harding, 2006). If adversity coincides with enhanced susceptibility during critical “windows” of development, it may alter the developmentally programmed phenotype, with potentially long-lasting deleterious consequences. The present study extends our previous work (McDonald et al., 2015b), which demonstrated that pCIH exposure results in sternohyoid muscle weakness in male and female rats, to illustrate that the airway dilator muscle appears to recover function with advancing age into adulthood. Our data show very little difference in sternohyoid (and diaphragm) muscle force and endurance at 16 weeks comparing sham and pCIH-exposed groups (both male and female rats). This is encouraging in some respects if the data here resemble potential outcomes of pCIH exposure in human infants. On the face of it, our data suggest that pCIH-induced muscle dysfunction is restricted to a developmental timeline spanning the early life to adolescent period (McDonald et al., 2015b), and this disability appears to wane with advancing age (this study), once normal oxygen homeostasis is returned.

Our study revealed that early life exposure to CIH increased the susceptibility of sternohyoid muscles to subsequent hypoxic exposure later in life. It is interesting to consider that no material sex differences were observed in our study. Exposure to p+aCIH decreased sternohyoid muscle force both in males and females, and the magnitude of the effect was similar in both sexes. The finding is consistent with our previous observation that pCIH exposure impairs male and female sternohyoid muscle force into adolescence (McDonald et al., 2015b). It is suggested that human males may be more vulnerable to adversity than their female counterparts; for example, newborn males are more at risk of developing respiratory disorders (including SIDS) than females (Mage and Donner, 2006). Our studies (McDonald et al., 2015b; this study) clearly illustrate, however, that early life exposure to CIH adversely affects male and female rat respiratory muscle in equal measure.

We observed differential effects comparing sternohyoid and diaphragm muscle responses to combined CIH exposure (p+aCIH). Sternohyoid and diaphragm have very different fiber type compositions during development (O'Connell et al., 2013) and into adult life (McMorrow et al., 2011); this may underlie, at least in part, differential susceptibility to CIH-induced dysfunction. Costello et al. (2008) examined the impact of altered maternal body condition on glucose uptake in muscles of adult offspring; while fast fiber vastus muscles showed structural alterations, the slow fiber soleus muscle was reportedly unaffected and therefore may be less susceptible due to a higher proportion of type 1 myofibers (Ward and Stickland, 1991; Dwyer et al., 1994). The fast fiber sternohyoid muscle appears to show increased vulnerability to hypoxic stress compared with diaphragm and this may have implications for the control of airway caliber *in vivo*. The sternohyoid and diaphragm have complementary respiratory functions, but a mismatch in the force-generating capacities of the two muscles could be detrimental to the control of airway patency, since a balance of forces dictates airway caliber on a breath-by-breath basis. Of note, we have previously reported increased susceptibility of sternohyoid compared with diaphragm in response to early life exposure to sustained hypoxia (Carberry et al., 2014).

The changes observed in sternohyoid muscle in this study may be associated with permanent alterations in gene expression orchestrated by hypoxic exposure during early life. Alterations in gene expression are regulated by graded changes in epigenetic factors such as the degree of DNA and histone methylation/acetylation and post-transcriptional and post-translational modifications. Of note, Nanduri and Prabhakar (2013) provides evidence that increased DNA methylation induced by neonatal IH is implicated in neonatal programming. Genetic variation may play a role in determining subsequent sensitivity to environmental stressors such as intermittent hypoxia. These and other potential mechanisms contributing to increased vulnerability of sternohyoid muscle to hypoxic stress would be interesting areas to explore in future studies.

Our studies also suggest that younger animals display a greater capacity for hypoxia-induced muscle plasticity compared with adult animals (Carberry et al., 2014; McDonald et al., 2014, 2015b; this study). Reeves and Gozal (2006) demonstrated that the magnitude of IH-induced respiratory plasticity is age-dependent with progressive reductions becoming apparent with advancing postnatal age. We have shown that the same CIH paradigm which caused force decline in neonates (that persisted for at least 3 weeks; McDonald et al., 2015b), caused little or no effect when exposures were applied in adulthood (McDonald et al., 2014) and this was confirmed by way of assessment of the effects of CIH on sternohyoid function in adult females in this study. Similarly, Skelly et al. (2011) reported increased tolerance to CIH in the sternohyoid of aged male rats compared with young adults (Skelly et al., 2012). It is also clear however that CIH-induced maladaptation is not strictly a developmental phenomenon, since it is established that more intense paradigms of CIH can induce alterations in muscle physiology during adulthood. As such, Skelly et al. (2012) reported upper airway muscle dysfunction in adult rats after 9 days of CIH (20 cycles of IH, 5% at nadir, 8 h a day), a paradigm different from that used in the present study (12 cycles of IH, 5% at nadir, 8 h a day). Indeed it is evident that the pattern, duration, and intensity of IH exposure are important determinants of CIH-induced muscle plasticity (Shortt et al., 2013). Evidence increasingly suggests that CIH generally has no major effect on sternohyoid, diaphragm or limb muscle fiber type expression or cross-sectional area (McGuire et al., 2002, McDonald et al., 2014, Skelly et al., 2012), though a reduction in fiber cross-section may contribute to sternohyoid weakness following pCIH (McDonald et al., 2015b). The structural phenotype of muscle is not easily altered by CIH, which may not be surprising given the metabolic cost of structural changes. Though the molecular mechanisms underpinning functional remodeling in respiratory muscles are unclear at this time they clearly warrant further investigation as the findings may have translational value. A recent study from our group implicates altered NADPH oxidase-dependent redox signaling in adult rat sternohyoid exposed to CIH (Williams et al., 2015). CIH stress is insufficient to cause overt oxidation of protein and lipids in respiratory muscle (McDonald et al., 2015b, Williams et al., 2015), but the ensuing modest redox stress which weakens muscle is amenable to antioxidant supplementation (Shortt et al., 2014, Skelly et al., 2012).

In summary, this study reveals increased susceptibility to hypoxia in male and female sternohyoid muscle (but not diaphragm) in adulthood that relates to antecedent intermittent hypoxia exposure during postnatal development, revealed following re-exposure to the stressor during later life. The CIH stress is itself apparently innocuous in adulthood, insofar as exposure in otherwise naïve animals (i.e., reared in normoxia) does not affect sternohyoid and diaphragm muscle function. Our data may have implications for the control of airway patency in humans who are exposed to intermittent hypoxia in early life (e.g., AOP, chronic lung disease). The pharyngeal dilator muscles play a pivotal role in the control of airway caliber. Upper airway muscle dysfunction increases the risk of upper airway collapse. As such, it is tempting to speculate that if airway dilator weakness is a consequence of CIH exposure in humans during early development (or at any time), there may be a greater propensity for airway collapse, which could trigger an inescapable cycle perpetuating obstructive airway conditions. We acknowledge however the need for cautious extrapolation of the findings of our study in an animal model to human disorders. Our study highlights the need for additional studies exploring the long-term consequences of early life hypoxia on respiratory system performance in adult life. Of note, neonatologists remain uncertain of optimal target oxygen saturation levels in preterm infants e.g., 86–90 or 91–95%. Interventional treatment in this setting, which in the majority of circumstances is an increase in oxygen delivery, probably results in periods of hypoxia alternating with hyperoxia. This would be an interesting model to explore in future studies.

## Author contributions

FM and KO designed the study. FM conducted experiments and analyzed data. FM and KO interpreted data sets and performed statistical analyses. All authors contributed to the writing of the manuscript.

## Funding

Funded by Health Research Board (Ireland). FM was funded by the School of Medicine and Medical Science, University College Dublin, Ireland.

### Conflict of interest statement

The authors declare that the research was conducted in the absence of any commercial or financial relationships that could be construed as a potential conflict of interest.
